# Oxidative damages in erythrocytes of patients with metabolic syndrome

**DOI:** 10.1007/s11010-013-1617-7

**Published:** 2013-03-21

**Authors:** A. Ziobro, P. Duchnowicz, A. Mulik, M. Koter-Michalak, M. Broncel

**Affiliations:** 1 Department of Environment Pollution Biophysics, Faculty of Biology and Environmental Protection, University of Lodz, 141/143 Pomorska St., 90-237 Lodz, Poland; 2 Department of Internal Diseases and Clinical Pharmacology, Medical University of Lodz, 1/3 Kniaziewicza St., 91-347 Lodz, Poland

**Keywords:** Metabolic syndrome, Erythrocyte, Oxidative damage, Cholesterol, Antioxidant enzymes, Glutathione, Lipid peroxidation

## Abstract

The aim of the study was to estimate the changes caused by oxidative stress in structure and function of membrane of erythrocytes from patients with metabolic syndrome (MS). The study involved 85 patients with MS before pharmacological treatment and 75 healthy volunteers as a control group. Cholesterol level, lipid peroxidation, glutathione level (GSH), and antioxidant enzyme activities in erythrocytes were investigated. The damage to erythrocyte proteins was also indicated by means of activity of ATPase (total and Na^+^,K^+^ ATPase) and thiol group level. The membrane fluidity of erythrocytes was estimated by the fluorescent method. The cholesterol concentration and the level of lipid peroxidation were significantly higher, whereas the concentration of proteins thiol groups decreased in the patient group. ATPase and GSH peroxidase activities diminished compared to those in the control group. There were no differences in either catalase or superoxide dismutase activities. The membrane fluidity was lower in erythrocytes from patients with MS than in the ones from control group. These results show changes in red blood cells of patients with MS as a consequence of a higher concentration of cholesterol in the membrane and an increased oxidative stress.

## Introduction

The metabolic syndrome (MS) was identified as a multiplex risk factor for cardiovascular disease which is one of the major causes of death. Overweight or obesity, abnormal lipid metabolism, hypertension, and insulin resistance are the major contributors in MS.

According to the International Diabetes Federation (IDF) new definition, a diagnosis of MS can be made when a person has central obesity (waist circumference >94 cm for Europid men and >80 cm for Europid women) and two out of four additional factors are present: raised triglyceride level ≥150 mg/dL or specific treatment for this lipid abnormality, reduced HDL cholesterol level <40 mg/dL for men and <50 mg/dL for women or specific treatment for this lipid abnormality, raised blood pressure (systolic ≥130 mm Hg or diastolic ≥85 mm Hg) or treatment of previously diagnosed hypertension and raised fasting plasma glucose ≥100 mg/dL (5.6 mM) or previously diagnosed type 2 diabetes [1].

The MS is an increasingly significant global health problem. The National Health and Nutrition Examination Survey 1999–2002 showed that almost 40 % of the U.S. adults were classified as having the MS [2]. The prevalence of MS among Polish adults is estimated at the level of 23 % (men) and 20 % (women) [3].

The MS is believed to be a consequence of a sedentary lifestyle, excessive intake of calories and physical inactivity, although genetic factors are also involved [4, 5].

The elevated oxidative stress, increased leptin and decreased adiponectin levels, the activation of inflammatory cytokines and prothrombotic mediators are associated with MS [6, 7]. The reduction of antioxidant enzyme activities and an increase in lipid peroxidation are results of a prolonged state of oxidative stress.

The following study aims to investigate the changes in the plasma membrane of erythrocytes and in the activities of antioxidant enzymes caused by the MS. Alterations in plasma lipid profile and oxidative stress can affect erythrocyte membranes and disturb the structure and function of cell membranes.

## Materials and methods

### Patients

The study involved 75 healthy individuals and 85 patients with MS diagnosed according to IDF definition. The exclusion criteria were as follows: (i) secondary dyslipidemia, (ii) any acute or chronic inflammatory processes, (iii) arterial hypertension, (iv) diabetes mellitus, (v) impaired renal or hepatic function, (vi) treatment with hypolipidemic, hypotensive, anticoagulant drugs, (vii) antioxidant therapy, (viii) abuse of alcohol or smoking cigarettes.

These experiments were conducted in accordance with ethical standards as formulated in Helsinki Declaration of 1975 (revised 1983); consent number 241/06/KB of Commission of Medical Research Ethics of Medical University of Lodz, Poland.

Blood from MS patients was obtained from the Department of Clinical Pharmacology, Medical University of Lodz. The samples from healthy volunteers were provided by the Central Blood Bank of Lodz. Blood was collected with anticoagulant (23 mM citric acid, 45.1 mM trisodium citrate, 45 mM glucose) in 5:1 ratio.

### Erythrocytes

Erythrocytes were obtained from blood by centrifugation (600×*g*, 10 min, 4 °C) and then washed three times with cold 0.9 % NaCl. They were examined immediately after isolation.

### Red cell membrane preparation

Erythrocyte membranes were prepared by the method of Dodge [8] with buffer modification. Erythrocytes were hemolyzed with 20 mM Tris–HCl buffer, pH 7.4, supplemented with 1 mM EDTA and 0.01 % PMSF on ice for 15 min. The erythrocyte membranes were centrifuged at 20,000×*g* for 5 min. The membranes were washed several times with the above-mentioned buffer until the “white ghost” (hemoglobin-free) state was attained. All buffers were cooled to 4 °C prior to use and the whole preparation procedure was conducted on ice.

The protein concentration was estimated according to the method of Lowry [9]. The concentration of protein in the sample was read from a calibration curve in the range 50–300 μg proteins/ml using albumin from bovine serum as the standard.

### Cholesterol concentration

The extraction of lipids from erythrocytes was carried out according to the method of Rodriguez-Vico [10] using chloroform/methanol mixture (2:1 v/v). The concentration of cholesterol was determined using Liebermann–Burchard reagent [11] and was expressed as milligrams of cholesterol per milliliter of packed cells (mg HC/ml packed cell).

### Peroxidation of lipids

The peroxidation of lipids was estimated by the measurement of compounds reacting with 2-thiobarbituric acid (TBA) according to the method of Stock and Dormandy [12]. The absorbance was read at 532 nm. The concentration of TBARS was calculated using a millimolar absorption coefficient for malondialdehyde, *ε* = 1.5 × 10^5^ mol^−1^ dm^3^ cm^−1^. The hemoglobin concentration was determined by Drabkin method [13]. Absorbance was read at 540 nm. The results were expressed as μmol TBARS/g Hb.

### ATPase activity

The activity of ATPase was measured by means of the method of Bartosz [14] based on the quantification of orthophosphate released from ATP during the incubation of erythrocyte membranes with medium (1 mmol/dm^3^ ATP, 10 mmol/dm^3^ MgCl_2_, 100 mmol/dm^3^ buffer Tris–HCl, pH 7.4 for total ATPase activity and additionally supplemented with 0.1 mmol/dm^3^ ouabain to verify activity of Na^+^/K^+^ ATPase). The concentration of orthophosphate released from ATP was determined in supernatant by the method of van Veldhoven and Mannaerts [15] and was read from a calibration curve in the range of 2–20 μM using KH_2_PO_4_ as the standard. The results were expressed as nmol orthophosphate/mg proteins × h. The activity of Na^+^ K^+^ ATPase was quantified as a difference between ouabain treated and non-treated membranes.

### Concentration of thiol groups and glutathione level

The level of thiol groups in erythrocyte membranes and of glutathione (GSH) in erythrocytes was estimated according to the method of Ellman with 5,5′-dithiobis-(2-nitrobenzoic acid) (DTNB). The concentration of –SH groups was calculated using a milimolar absorption coefficient for 2-nitro-5-thiobenzoate (NTB^−^), *ε*_412_ = 13.6 mmol^−1^ dm^3^ cm^−1^ [16]. The results were expressed as μmol –SH/mg proteins or μmol GSH/ml packed cell.

### Fluidity of erythrocyte membranes

The fluidity of erythrocyte membranes was measured by means of fluorescent anisotropy using two fluorescent probes: 1,6-diphenyl-1,3,5-hexatriene (DPH) and 1-[4′-(trimethylammonium)phenyl]-6-phenyl-1,3,5-hexatriene (TMA–DPH). DPH is localized in hydrophobic region near the center of the bilayer, while TMA–DPH is incorporated into the polar region of the erythrocyte membrane. The increase in fluorescence anisotropy indicates that the membrane becomes more rigid (less fluid) [17].

Fluorescence anisotropy measurements were carried out with an LS-50B fluorescent spectrometer (Perkin–Elmer, UK). The excitation and emission wavelengths were 348 and 426 nm, respectively. The cuvette holder was temperature controlled (37 °C). Erythrocyte membranes were diluted with buffered saline to protein concentration of 100 μg/ml. Final concentration of fluorescent probes was 1 μmol/l.

The fluorescence anisotropy (*r*) of the samples was calculated by the fluorescence data manager program using the following equation:

$$ r \, = \, (I_{\text{vv}} - GI_{\text{VH}} )/\left( {I_{\text{VV}} + \, 2GI_{\text{VH}} } \right) $$

where *I*_VV_ and *I*_VH_ were the vertical and horizontal fluorescence intensities, respectively, to the vertical polarization of the excitation light beam. The factor *G* = *I*_HV_/*I*_HH_ corrects the polarizing effects of the monochromator.

### Antioxidant enzymes

Superoxide dismutase (SOD) activity was estimated according to the method of Misra and Fridovich [18] with epinephrine. The result was expressed as U/g Hb.

The activity of catalase (CAT) was measured by means of the method of Aebi [19]. CAT activity was expressed as U/g Hb.

The glutathione peroxidase (GPx) activity was estimated according to the method of Rice-Evans et al. [20]. The result was expressed as U/mg Hb.

### Statistical analysis

The results are presented as mean ± SD. Differences between groups were assessed by an unpaired Student’s *t* test. The statistical analysis was performed using STATISTICA^©^ ver 9.0 PL software (StatSoft Inc., Tulsa, USA).

## Results

The study cohort included 85 patients (37 men, 48 women). The control group included 75 healthy volunteers (33 men, 42 women). Patients with MS had significantly higher waist circumference, BMI values, levels of total cholesterol (increase about 21 %), LDL-C (increased by 23 %), triglycerides (increased by 267 %), glucose (increased by 16 %), systolic and diastolic pressure, and significantly lower level of HDL-C (decreased by 26 %) when compared to healthy controls. Level of CRP did not differ significantly between groups (Table 1).

**Table 1:** Characteristics of control group and patients with MS

Parameter	Control group	Patients with MS	*p*
*n*	75	85	
Age	50.3 ± 8.2	55.9 ± 7.4	
Men/women	33/42	37/48	
Waist circumference (cm)	75.7 ± 10.0	98.1 ± 18.8	0.001
Body weight (kg)	67.0 ± 7.9	90.7 ± 12.5	0.001
BMI (kg/m^2^)	24.9 ± 2.2	30.5 ± 3.4	0.001
Systolic pressure (mmHg)	117.2 ± 11.7	132.7 ± 7.4	0.001
Diastolic pressure (mmHg)	71.2 ± 6.9	79.2 ± 6.7	0.001
hs-CRP (mg/l)	1.6 ± 1.4	2.7 ± 2.3	NS
Glucose (mg/dl)	82.3 ± 9.2	95.7 ± 10.5	0.001
Total cholesterol (mg/dl)	199.5 ± 24.5	242.2 ± 36.8	0.001
LDL-C (mg/dl)	120.7 ± 19.8	148.8 ± 35.2	0.01
HDL-C (mg/dl)	58.7 ± 10.8	43.8 ± 9.8	0.001
TG (mg/dl)	96.5 ± 29.4	258.2 ± 94.9	0.001

*hs-CRP* high sensitivity C-reactive protein,* LDL-C* low density lipoprotein cholesterol,* HDL-C* high density lipoprotein cholesterol,* TG* triglycerides,* NS* non-statistical significant

Patients with MS had significantly higher levels of cholesterol (about 45 %) in erythrocyte membrane in comparison to healthy controls (Table 2). The increase in cholesterol concentration in plasma, especially LDL-C, led to increase in the accumulation of cholesterol in erythrocyte membrane.

**Table 2:** Cholesterol concentration, level of lipid peroxidation and membrane fluidity in erythrocytes for control and MS patients groups

Parameter	Control group	MS patients	*p*
Total cholesterol content (mg CH/ml packed cell)	2.82 ± 0.64 (*n* = 56)	4.09 ± 1.19 (*n* = 80)	0.001
Lipid peroxidation (µmol TBARS/g Hb)	0.329 ± 0.078 (*n* = 73)	0.453 ± 0.140 (*n* = 82)	0.001
TMA–DPH fluorescent anisotropy (r)	0.308 ± 0.019 (*n* = 22)	0.339 ± 0.047 (*n* = 28)	0.01
DPH fluorescent anisotropy (r)	0.271 ± 0.022 (*n* = 22)	0.297 ± 0.032 (*n* = 28)	0.005

In erythrocytes from MS patients, higher level of lipid peroxidation (about 37 %) in comparison to healthy controls was observed (Table 2). This might be a consequence of the increase in the level of oxidants caused by redox imbalance in cells.

Patients with MS demonstrated substantially lower membrane fluidity (about 10 % higher fluorescent anisotropy values for both probes) than the control group (Table 2). Membrane fluidity depends on the composition of lipids and proteins. Higher level of cholesterol decreases membrane fluidity, especially in polar region of the monolayer. Lipid peroxidation also disturbs membrane fluidity. Incorporation of polar group (ketone, aldehyde, hydroxyl, superoxide) to double bond in the hydrophobic region leads to impairment of the structure of membrane and affects other parameters.

The concentration of thiol groups in erythrocyte membrane observed for patients with MS was significantly lower (about 16 %) than in the control group (Table 3). The altered level of thiol groups may be a result of the modification of protein conformation or directly damage caused by free radicals. The decrease in total ATPase activity (8.4 %) and Na^+^ K^+^ ATPase activity (26 %) was observed in erythrocytes from MS patients in comparison with the control group (Table 3). Lower activity of ATPase may be caused by change in membrane viscosity (fluidity) or by direct protein damage by free radicals or other products emerging during lipid peroxidation.

**Table 3:** Thiol groups and ATPase activity in erythrocyte for control and MS patient groups

Parameter	Control group	MS patients	*p*
Total thiol groups content (nmol –SH/mg proteins)	81 ± 25 (*n* = 34)	68 ± 20 (*n* = 78)	0.01
Total ATPase activity (nmol Pi/mg protein × h)	536.8 ± 134.5 (*n* = 52)	491.6 ± 116.8 (*n* = 70)	0.05
Na^+^, K^+^ ATPase activity (nmol Pi/mg protein × h)	249.5 ± 86.7 (*n* = 52)	185.0 ± 68.5 (*n* = 70)	0.001

The activity of GPx for patients with MS was significantly lower (13 %) than that observed in the control group. No significant changes in activities of SOD or CAT were observed. The concentration of GSH was significantly lower (8 %) in patients with MS in comparison to the control group (Table 4).

**Table 4:** Level of GSH and activities of antioxidant enzymes in control and MS patient groups

Parameter	Control group	MS patients	*p*
GSH concentration μmol GSH/ml packed cell	1.37 ± 0.37 (*n* = 75)	1.26 ± 0.29 (*n* = 74)	0.05
Glutathione peroxidase (GPx) activity U/g Hb	20.91 ± 4.65 (*n* = 65)	18.25 ± 4.72 (*n* = 82)	0.001
Catalase (CAT) activity U/mg Hb	261.1 ± 64.2 (*n* = 70)	261.6 ± 60.3 (*n* = 80)	NS
Superoxide dismutase (SOD) activity U/g Hb	2856 ± 644 (*n* = 73)	2770 ± 621 (*n* = 82)	NS

*NS* Non-statistical significant

Higher level of lipid peroxidation and lower activity of GPx may suggest that patients with MS demonstrate higher level of oxidative stress than the control group.

## Discussion

The oxidative stress plays an important role in dysfunction of endothelial pathogenesis, hypertension, inflammation conditions, atherosclerosis [21], hypercholesterolemia [22], and diabetes [23]. An intensification of oxidative stress in patients with MS was previously reported [24]. Shim et al. [25] observed an important growth in the number of neutrophils, lymphocytes, monocytes, and eosinophils, which generate a large amount of reactive oxygen species (ROS) after stimulation in patients with MS. As a result of higher generation of ROS, the damage of lipids, proteins, and nucleic acids is observed in cells. Mittal and Kant [26] observed an increase of concentration of malondialdehyde in erythrocytes from postmenopausal woman suffering from obesity, which directly correlates with body mass index. An increase in the concentration of TBARS and conjugated dienes in erythrocytes derived from patients diagnosed with type-2 hypercholesterolemia was reported [22]. Plasma membrane lipids are documented to be the major targets for ROS in erythrocytes. In this study, the level of lipid peroxidation (TBARS) was significantly higher in erythrocytes from patients with MS in comparison to the control group. This result suggests disturbance of pro- and antioxidant balance.

The major disturbance in MS is an abnormal lipid metabolism: elevated plasma triglycerides, increased level of LDL, and decreased level of HDL. The altered membrane microviscosity reflects the changes of membrane lipid composition resulting from intensive exchange between circulating and membrane lipids, as well as from abnormal cellular lipid synthesis and metabolism [27]. The LDL of blood plasma was capable of exchanging the cholesterol with erythrocyte membranes [28]. The concentration of total cholesterol in erythrocytes, derived from MS patients, was significantly higher when compared to that of the control group. These results confirm data obtained for erythrocytes from patients with hypercholesterolemia [22, 29]. Hypercholesterolemic people demonstrate a higher erythrocyte membrane cholesterol/phospholipid ratio. This alteration of the lipid membrane correlates inversely with the plasma concentration of HDL-cholesterol [30]. The higher level of cholesterol, especially higher level of LDL-C, and raised production of ROS may lead to intensification of atherosclerosis processes.

Changes in cholesterol content and level of lipid peroxidation affect the membrane fluidity. The decrease in plasma membrane fluidity was observed as a result of the increase in the concentration of cholesterol in plasma membrane [31]. A significant drop in membrane fluidity in erythrocytes from patients with type-2 hyperlipidemia together with the increase in cholesterol concentration was previously reported [22]. In this study, the increase in fluorescent anisotropy for both fluorescent probes indicates the decrease in fluidity of plasma membrane in hydrophilic and hydrophobic regions. The decrease in membrane fluidity may further lead to the decrease in the ability of erythrocytes to change shape and finally to disorder of the blood flow.

The membrane lipid composition and elevated oxidative stress may contribute to the changes in the activity of the main membrane enzymes. A higher cholesterol concentration in the membrane was documented to decrease activities of Ca^2+^ Mg^2+^ ATPase and Na^+^ K^+^ ATPase, and disturbs ion transport [32, 33]. The activity of Na^+^ K^+^ ATPase diminished in persons with familial hypercholesterolemia [21]. The decrease in Na^+^ K^+^ ATPase activity can be also associated with diabetes [34]. The reactive form of oxygen, nitrogen, and chlorine may modify membrane protein and may inhibit enzymes, including ATPases [35, 36]. Also products of lipid peroxidation might contribute to disturbance of ATPase activity. In the present study, both the total ATPase and Na^+^ K^+^ ATPase activities decreased in comparison to those in the control group. The decrease in Na^+^ K^+^ ATPase activity disturbs not only transport of Na^+^ and K^+^ ions across membrane, but also can modify resting potential of membrane and volume of cell. In addition, Na^+^ gradient is also used as a source of energy by certain carrier processes, e.g., Na^+^-glucose symport. The decrease in Na^+^ K^+^ ATPase activity may therefore lead to the development of cardiovascular complications.

GSH plays a key role in cellular homeostasis. It is a major factor in regulation of cell life, proliferation, and death. GSH was also involved in cell defense against prooxidants, both directly as low-weight antioxidants and as a cofactor for GPx. In the present study, the decrease in GSH concentration was observed. Substantial decrease in the GSH concentration and the increase in GSSG/GSH ratio in patients with hypertension, dyslipidemia, MS, and obesity were reported [37, 38]. Similar changes in plasma from patients with type 2 diabetes were also noticed [39].

The decrease in concentration of thiol groups in erythrocytes membranes from type-2 hypercholesterolemic patients was reported earlier [22]. Thiol groups play an important role in folding and stability of proteins, including enzymes, and in the modulation of a variety of activities of the cell membrane, including transport processes across the membrane. Thiol groups from membrane proteins are liable to modifications caused by oxidants or alkyl agent. Changes in level of thiol groups are a result of the oxidative damage to membrane proteins or changes in structure of proteins. In this study, patients with MS demonstrated lowered contents of proteins thiol groups compared to the healthy group.

The antioxidant enzymes are major parts of cell defense against ROS. In the present research, the GPx activity was significantly decreased, while activities of CAT or SOD were similar to those in the healthy group. Karaouzen et al. [40] found correlation between activities of antioxidant enzymes and aging in healthy and obese men. Activities of SOD, CAT, and GPx were higher in healthy young men when compared to those in older men. Activities of SOD and CAT were enhanced in obese young men and reduced in older men, and activity of GPx was lower in both groups. Mansengo et al. [38] documented lower activities of CAT and GPx in patients with hypercholesterolemia and hypertension, while activity of SOD was only reduced in patients with hypertension. Cardona et al. [37] noticed the increase in CAT activity and the decrease in GPx activity in patients with hypertriglyceridemia, regardless of MS. Also Cardona et al. [41] observed the decrease in GPx activity, but no changes in activities of SOD or CAT in blood from patients with MS were observed when compared to those in the control group. It seems that activities of antioxidant enzymes depend on age of subjects, tissue, associated illness, and dietary habit.

## Conclusions

The above-presented results point to some essential changes in the erythrocyte plasma membranes. The following changes occur in plasma membrane lipids: the increase in cholesterol concentration and in levels of lipid peroxidation as well as the decrease in plasma membrane fluidity. Collected data also demonstrate changes in functions of plasma membrane proteins: the decrease in concentration of thiol groups and the inhibition of ATPase activity.

Antioxidant enzymes represent an important part of the antioxidant defense system of aerobic cells. The decrease in antioxidant levels and increase in lipid peroxidation resulted in oxidative stress in blood of patients with MS.

The MS is not a disease in the strict sense of this word. It is a metabolic disorder which has multi-factor pathology. Changes observed in erythrocytes occur as a result of all disorders connected to abnormal metabolism of lipids, diabetes and hypertension.

## Abbreviations

MS: Metabolic syndrome
LDL: Low-density lipoprotein
HDL: High-density lipoprotein
TBA: Thiobarbituric acid
TBARS: Thiobarbituric acid reactive species
SOD: Superoxide dismutase
CAT: Catalase
GPx: Glutathione peroxidase
ROS: Reactive oxygen species
GSH: Reduced glutathione
GSSG: Oxidized glutathione
